# Quality of Life, Social Desirability and Their Relationship in Opium Addicted Persons in Southeast of Iran

**DOI:** 10.5539/gjhs.v6n3p97

**Published:** 2014-02-22

**Authors:** Mansour Arab, Mehri Kohan, Hadi Ranjbar, Nanaz Arab, Masoud Rayani, Salehe Sadat Mirrashidi, Hossein Rafiei, Masoud Amiri

**Affiliations:** 1 Department of Medical- Surgical Nursing, Razi School of Nursing and Midwifery, Kerman University of Medical Sciences, Kerman, Iran; 2 Neuroscience Research Center, Kerman University of Medical Sciences, Kerman, Iran; 3 School of Pharmacy, Kerman University of Medical Science, Kerman, Iran; 4 Department of Health, Razi School of Nursing and Midwifery, Kerman University of Medical Sciences, Kerman, Iran; 5 Department of Intensive and Critical Care, School of Nursing and Midwifery, Shahrekord University of Medical Sciences, Shahrekord, Iran; 6 Social Health Determinants Research Center, Shahrekord University of Medical Sciences, Shahrekord, Iran

**Keywords:** quality of life, social desirability, opioid addicted, SF-36, MC_SDS, Iran

## Abstract

**Background and Aim:**

Addiction leads to many problems which may adversely affect addicted people, their families and impose health care agencies with many challenges. This study aimed to examined quality of life (QoL), social desirability and their relationship among opium addicted persons in southeast of Iran.

**Material and Methods:**

In a cross-sectional study conducted from September 2012 to January 2013, 123 addicted people were studied. Date collection tools were; checklist of demographic data, Iranian version of the 36-item short form QoL (SF-36) and Marlowe-Crowne Social Desirability Scale (MC-SDS).

**Results:**

While mean score of QoL was 60.4±29.5, mean score of social desirability were 14.2±3.7. Low, moderate and high levels of social desirability were observed in 4.9%, 90.2% and 4.9% of participants, respectively. Pearson’s correlation were not significant between mean score of social desirability and mean score of QoL (p=0.969, r=0.004).

**Conclusion:**

Addicted participants of present study showed a moderate level of QoL and social desirability, without any significant relationship between QoL and social desirability. Further research is suggested in addicts with social and cultural differences.

## 1. Introduction

Although opium has been used for medical purposes since long time ago (Nakhaee, 2009); nowadays, substance abuse of opium is one of the most complicated health problems worldwide which has been resulted many challenges for health care agencies (Bashardoost & Tirani, 2005; Najafi, 2009; Ziaaddini, 2006; Ziaaddini & Ziaaddini, 2005). Due to a very long border with the world’s largest opium producer, Afghanistan (Meysamie, 2009; Parvizy, 2005; Razzaghi, 2006), obtaining opium in Iran is too easy (Meysamie et al., 2009). Opium is the most common type of substance abuse in Iran (Aghaee-Afshar, 2008; Karbakhsh & Salehian Zandi, 2007; Nemati, 2010). Research has shown that the prevalence of opium addiction is approximately between 2 and 14.6 percent in different social groups of Iran (Ahmadi & Hasani, 2003; Mohammad Poorasl, 2007; Rajabizade, 2004; Ziaaddini & Ziaaddini, 2005). There is a very common belief in old Iranians that the use of opium could reduce aches and pains and may even decrease the rate of cardiovascular diseases (Karbakhsh & Salehian Zandi, 2007; Rafiei, 2012).

Quality of life (QoL) could be a key quality indicator in the evaluation of health care systems (De Maeyer, 2010; Hojjati, 2012), and opium addiction may affect on QoL (Hojjati et al., 2012). Due to importance of addiction worldwide, QoL among opiate-dependent individuals has been received considerable attention in recent researches. For example, Bizzari and colleagues (2005) have compared QoL of 57 patients with opioid dependence alone, 41 with opioid dependence and a psychiatric disorder and 45 healthy persons in Italy. They reported that patients with opioid dependence and a psychiatric disorder have had worse QoL compared to patients without psychiatric disorder. They also found that both groups of patients had poorer QOL in the physical, psychological, and social domains in comparison with healthy persons. In another study, Ponizofsky and Grinshpoon (2007) have studied QoL of heroin users who starting a maintenance treatment program using methadone versus buprenorphine. They reported that both methods of maintenance had improved all domains of QoL of heroin users. They also showed that users whom were maintained on methadone have had an earlier onset in improving QoL than patients with buprenorphine maintenance. In 2005, Giacomuzzi and colleagues have examined the effects of gender on QoL of 103 opioid users. They did not find any significant difference in terms of QoL and physical symptoms between addicted men and women. Furthermore, another important issue in improving QoL of opium-addicted patients is social desirability (Hojjati, 2012). Poor social desirability may also adversely affect on QoL of opium-addicted patients.

Since there were a few studies on these topics, in the present study, QoL, social desirability and their relationship in opium-addicted persons who admitted to a maintenance treatment center have been studied in Kerman, southeast of Iran.

## 2. Materials and Methods

In a cross-sectional study conducted from September 2012 to January 2013 in Kerman, southeast of Iran. Convenience samples of 123 people who addicted to opium and admitted to a maintenance treatment center were studied. The written permission was obtained from deputy of research and also the Ethics’ Board of the Kerman University of Medical Sciences. Inclusions criteria were: addiction to opium (more than one year) and ability to answer the provided questionnaire. Exclusion criteria were addiction to other forms of substances and history of mental disorder. Questionnaires were handed out by the second researcher, along with a letter providing information about the aims of the study. Participants answered individually and returned the questionnaire to the researcher. Written consent letters were filled in by all respondents. All participants were promised that all data would remain anonymous, kept confidential and be stored safely.

The various instruments were used for data collection: 1) checklist of demographic data (including age, sex, marital status, job, level of education, type of opium used, age at first use, years of regular use and history of withdrawal), 2) Iranian version of the 36-item short form QoL (SF-36) (This form is a generic multidimensional instrument consisting of eight multi-item components representing physical functioning, role functioning physical, bodily pain, general health perceptions, vitality, social functioning, role functioning emotional and mental health (Juenger, 2002) and 3) Marlowe-Crowne Social Desirability Scale (MC-SDS) (this scale consist of 33 statement to which respondents are asked to answer true (T)-or-false (F). The (T) response was given the value 1 and the (F) response was given a value of zero. The total score on the test was the sum of all scores. Score between 0-8 indicates low social desirability, 9-19 refers to moderate social desirability and 20 – 33 means high level of social desirability (Crowne & Marlowe, 1960; Vu, 2010).

Continuous variables were presented by mean and standard deviation. Pearson’s correlation coefficient, independent t-test and ANOVA were applied for correlation and comparison. SPSS software (version 18.0) was used and p-value less than 0.05 were considered as statistically significant.

## 3. Results

Of 123 addicted persons, 101 (82.1%) persons were men. The mean age of participants was 34.8±9.3 years, 63.6% of them were married, the mean duration of addiction was 11.8±8.1 year, and the mean age of initiating opium use was 21.5±5.8 year. Table 1 shows more information about demographic characteristics.

**Table 1:** Participants demographic characteristics’

Demographic characteristics’		Number and present
Gender	Male	101(82.1%)
	female	22 (17.9%)
Marital status	Married	78 (63.4%)
	single	38 (30.9%)
	Divorced	3 (2.4%)
	Widow	4 (3.3%)
Education	Primary school	33 (26.8%)
	High school	79 (64.3%)
	2 years of college	7 (5.7%)
	Graduate	3 (2.4%)
	Post graduate	1 (0.8%)
Cause of Addiction	Curiosity	15 (12.2%)
	Friends	51 (41.5%)
	Treatment of disease	11 (8.9%)
	Relief of pain	25 (20.3%)
	Other causes	21 (17.1%)
Mean years of addiction	1- 4 year	20(17.1%)
	4 – 7 year	23(19.7%)
	7- 11 year	21(17.9%)
	11- 15 year	10(8.5%)
	15- 19 year	17(14.5%)
	19- 23 year	17(14.5%)
	More than 23 year	9(7.7%)

Mean score of social desirability were 14.2±3.7. Low, moderate and high levels of social desirability were observed in 4.9%, 90.2% and 4.9% of participants, respectively (Table 1).

Mean score of QoL was 60.4± 29.5. Higher score was related to sub scale of role functioning physical (128.4± 18.4) and lower score was related to sub scale social functioning (17.64± 31.4) (Table 2).

**Table 2:** Mean score of QoL subscale

	Mean Score
Physical Functioning	128.4±18.4
Role-Physical	57.60±28.30
Bodily Pain	52.4±36.59
General Health	49.9±19.85
Vitality	41.50±18.11
Social Functioning	17.64±31.4
Role-Emotional	117.8±113.8
Mental Health	41.4±27.54
Total Score	60.4±29.5

Pearson’s correlation were not significant between mean score of social desirability and mean score of QoL (p=0.969, r=0.004) as well as mean score of social desirability and mean score of each 8 sub scales of QoL (Table 3).

**Table 3:** Participants response to questions of social desirability questionnaire

Question Number	Number of (F) responses	Number of (T) responses
1- Before voting I thoroughly investigate the qualifications of all the candidates.	90 (73.2%)	33 (26.8%)
2- I never hesitate to go out of my way to help someone in trouble.	41(33.3%)	82 (66.7%)
3- It is sometimes hard for me to go on with my work if I am not encouraged.	79 (64.2%)	44 (35.8%)
4- I have never intensely disliked anyone.	73 (59.3%)	50 (40.7%)
5- On occasion I have had doubts about my ability to succeed in life.	89 (72.4%)	34 (27.6%)
6- I sometimes feel resentful when I don’t get my way.	98 (79.7%)	25 (20.3%)
7- I am always careful about my manner of dress.	100 (81.3%)	23 (18.7%)
8- My table manners at home are as good as when I eat out in a restaurant.	70 (56.9%)	53 (43.1%)
9- If I could get into a movie without paying and be sure I was not seen I would probably do it.	24 (19.5%)	99 (80.5%)
10- On a few occasions, I have given up doing something because I thought too little of my ability.	72 (58.5%)	51 (41.5%)
11- I like to gossip at times.	50 (40.7%)	73 (59.3%)
12- There have been times when I felt like rebelling against people in authority even though I knew they were right.	51 (41.5%)	72 (58.5%)
13- No matter who I’m talking to, I’m always a good listener.	93 (75.6%)	30 (24.4%)
14-I can remember “playing sick” to get out of something.	27 (22%)	94 (78%)
15-There have been occasions when I took advantage of someone.	28 (22.8%)	95 (77.2%)
16- I’m always willing to admit it when I make a mistake.	91 (74%)	32 (26%)
17-I always try to practice what I preach.	96 (78.9%)	26 (21.1%)
18- I don’t find it particularly difficult to get along with loud mouthed, obnoxious people.	55 (44.7%)	68 (55.3%)
19- I sometimes try to get even rather than forgive and forget.	44 (35.8%)	79 (64.2%)
20-When I don’t know something I don’t at all mind admitting it.	48 (39%)	75 (61%)
21-I am always courteous, even to people who are disagreeable.	95 (77.2%)	28 (22.8%)
22-At times I have really insisted on having things my own way.	77 (62.6%)	46 (37.4%)
23- There have been occasions when I felt like smashing things.	47 (38.2%)	76 (61.8%)
24-I would never think of letting someone else be punished for my wrongdoings.	91 (74%)	32 (26%)
25-I never resent being asked to return a favor.	102 (82.9%)	21 (17.1%)
26-I have never been irked when people expressed ideas very different from my own.	99 (89.5%)	24 (19.5%)
27-I never make a long trip without checking the safety of my car.	95 (77.2%)	28 (22.8%)
28-There have been times when I was quite jealous of the good fortune of others.	33 (26.8%)	90 (73.2%)
29-I have almost never felt the urge to tell someone off.	79 (64.2%)	44 (35.8%)
30-I am sometimes irritated by people who ask favors of me.	38 (30.9)	85 (69.1%)
31-I have never felt that I was punished without cause.	77 (62.6%)	46 (37.4%)
32- I sometimes think when people have a misfortune they only got what they deserved.	32 (26%)	91 (74%)
33-I have never deliberately said something that hurt someone’s feelings.	91 (74%)	32 (26%)

**Table 4:** Correlation between QoL score and Social desirability score

	Social desirability score		
	Number	Correlation	P value
Physical Functioning	123	-.035	.701
Role-Physical	123	-.035	.701
Bodily Pain	123	-.035	.701
General Health	123	-.035	.701
Vitality	123	-.035	.701
Social Functioning	123	-.035	.701
Role-Emotional	123	-.035	.701
Mental Health	123	-.035	.701
Total Score	123	-.035	.701

## 4. Discussion

Nowadays, opium addiction considerably affects on Iranian health care system. This study has considered QoL, social desirability and their relationship in opium-addicted persons who admitted to a maintenance treatment center have been studied in Kerman, southeast of Iran. Findings showed that addicted participants had moderate level of QoL.

Xiao and colleagues (2010) have studied the QoL of outpatients in methadone maintenance treatment in china. Similar to our finding, they also reported that addicted persons have moderate level of QoL which has been improved after methadone maintenance treatment. In another study, Hoseinifar and colleagues (2011) has compared QoL of addicted and non- addicted in Iran. In contrast with our findings, they found that addicted people lived in the worse condition than non-addicted persons. They also reported that addicted people needed more help and support from society. The observed difference between studies might be related to the difference in the place of research with different socioeconomic status. Indeed, Tehran with 12 million citizens as the biggest and more expensive city of Iran, has a good and high quality life than other cities of Iran and therefore more necessity to earn more income. It is obvious that it is very hard for an addicted person to acquire enough income to live in Tehran, which this low income in turn could affect on addicted people’s QoL.

Our findings showed that most of addicted people (90.2%) had moderate level of social desirability. Similar to our finding, Hojjati and colleagues (2012) reported that many of Iranian addicted persons (about 70%) had moderate level of social desirability. Furthermore, in Iran, there is a substantial negative attitude towards addiction and addicted persons. Addicted people usually have many problems such as job finding, marriage and obtaining vehicle driving license (Karbakhsh & Salehian Zandi, 2007). That’s why a large number of Iranian addicted people tend to re-consumption opium after detoxification programs (Shargh, 2011; Taghva, 2009; Mirzaei, 2010). Shargh and colleagues (2011) reported that one of the most common problems resulting in re-consumption among addicted persons is poor social acceptance of this group. Therefore, to improve the situation of addicted people, Iranian health care agencies should pay more attention to social dimension of addicted’s life.

No significant relationship was found between QoL and social desirability in this study. There was only one study in Iran on social acceptance and relationship with QoL of addicted persons referred to addiction centers in Golestan in 2012 (Hojjati, 2012). The mean score of QoL and social desirability of addicted people were 24.0± 1 and 13.0± 3, respectively. Although mean score of QoL and social desirability were less than our study; however, in contrast to our findings, they reported positive relationship between QoL and social desirability.

### 4.1 Limitation

The respondents were predominantly male, which limits the generalisability of the results for female addicts. As this study was based on a convenient sample and the participation was voluntary, there might have been a selection bias that affected the possibility to generalize the results to all addicted persons. Furthermore, use of the self reported questionnaires may have led to an overestimation of some of the findings because of the variance that is common in different methods.

## 5. Conclusion

Addiction has many problems which could adversely affect addicted people, their families and may impose health care agencies with many challenges. Addicted participants of present study showed a moderate level of QoL and social desirability, without any significant relationship between QoL and social desirability. Further researches will reveal the potential effects of social and cultural differences.
